# Increasing the digital repository of DNA barcoding sequences of sand flies (Psychodidae: Phlebotominae)

**DOI:** 10.1590/0074-02760190208

**Published:** 2019-11-28

**Authors:** Magdalena Laurito, Iliana M Ontivero, Walter R Almirón

**Affiliations:** 1 Universidad Nacional de Córdoba, Centro de Investigaciones Entomológicas de Córdoba, Facultad de Ciencias Exactas, Físicas y Naturales, Córdoba, Argentina; 2 Consejo Nacional de Investigaciones Científicas y Técnicas, Instituto de Investigaciones Biológicas y Tecnológicas, Córdoba, Argentina

**Keywords:** DNA barcoding, species delimitation, Evandromyia cortelezzii, Migonemyia migonei

## Abstract

Sand fly identification is complex because it depends on the expertise of the taxonomist. The females show subtle morphological differences and the occurrence of the species complexes are usual in this taxon. Therefore, a fragment of the cytochrome *c* oxidase subunit I (*COI*) gene is used for taxon barcoding to resolve this kind of problem. This study incorporates barcode sequences, for the first time, for *Evandromyia cortelezzii* and *Migonemyia migonei* from Argentina. The nucleotide sequence divergences were estimated to generate a neighbour-joining (NJ) tree. The automatic barcode gap discovery (ABGD) approach was employed to find the barcode gaps and the operational taxonomic unit (OTU) delimitation. Other species of the subtribe were included. The frequency histogram of divergences showed a barcoding gap. The ABGD analysis identified 14 operational taxonomic units (OTUs) from 13 morphological species. Sequences of *Ev. cortelezzii* and *Mg. migonei* formed well supported clusters and were diagnosed as primary species. These sequences are useful tools for molecular identification of the sand flies of the New World.

Phlebotominae is a subfamily of Psychodidae that is widely distributed with greater diversity in the tropical and subtropical regions.^1^ Until 2004, the southernmost distribution limit of Phlebotominae was 31º35′S-60º17′W, Entre Ríos Province, Argentina.^2^ ^,^ ^3^ Since then, several new records began to provide evidences regarding the expanding range of distribution of Phlebotominae to higher latitudes in Córdoba Province, Argentina. Salomón et al.^3^ recorded the specimens of *Migonemyia migonei* at 30º54′S-62º18′W in 2004. Visintin et al.^4^ reported specimens of the *Cortelezzii* complex at 30º51′S-62º56′W in 2012 and Ontivero et al.^5^ detected the southernmost record of both species in Córdoba City at 31º23′S-64º04′W during 2015-2016. The first autochthonous and the most southerly case of Tegumentary Leishmaniasis (TL) has been reported from the Córdoba Province in 2014.^5^

As not all sand fly species are vectors for pathogens, the accurate species identification during an entomological surveillance is needed to predict possible transmission foci. The use of morphological keys for the identification of Phlebotomine species depends on the expertise of the taxonomist. To accurately identify females of *Mg. migonei*, e.g. dissection of the last abdominal segments must be done to see the stalk-shaped spermathecal.^6^ The subtle morphological differences between females or males and the occurrence of species complexes also make species identification difficult. In the Cortelezzii complex^7^ females of *Evandromyia cortelezzii* and *Ev. sallesi* are difficult to differentiate because they share diagnostic features of the length of the clypeus and the spermathecal duct. The species can only be distinguished using male characters: *Ev. cortelezzii* has a hood-shaped paramere and a gonocoxite with a basal tuft of four long setae, and *Ev. sallesi* has the paramere without an apical hood-shaped structure and the gonocoxite has a basal tuft of five long setae.^6^ A fragment of the cytochrome *c* oxidase subunit I (*COI*) mitochondrial gene has been widely used in molecular taxonomy to resolve these kinds of issues.^8^ Even if several studies have employed *COI* barcode sequences to identify Phlebotominae species and revealed species complexes in America,^9^ ^,^ ^10^ ^,^ ^11^ (among others) its use can be problematic in closely related species because of incomplete lineage sorting and introgression events.^12^ The strategies designed to delimit species boundaries have been developed, among them is clustering, which does not require a *priori* definitions of species.^13^ Even if more than 220 sand fly species with barcode sequences are available in digital repositories, additional sequences are needed to enhance the efficiency of molecular identification. The present study incorporates, for the first time, barcode sequences of *Ev. cortelezzii* and the first sequence for *Mg. migonei* from Argentina and validates *COI* as an effective marker for taxonomic identification in sandflies because it shows consistent differences between closely related species.

Sand fly adults were captured with mini CDC light traps located at three sites: the Bajo Grande sewage treatment plant (31º23′38″S-64º04′36″W), the San Martín Urban Natural Reserve (31º21′44″S-64º15′47″W), and the Zoo Garden (31º25′34″S-64º10′33″W), once a week actively from 5 pm to 10 am, during March and April, 2018. The sand flies collected were killed by freezing and were stored at -20ºC in 80% ethanol until processed. Species identification was based on the structures of the head and internal and external genitalia of females and males, respectively, which were mounted on the microscope slides using the taxonomic key of Galati.^6^ Male sand fly legs (between two and six, according to availability), thorax, and abdomen were separated before morphological preparations for subsequent molecular procedures. Voucher specimens were deposited in the collection of the Centro de Investigaciones Entomológicas de Córdoba, Argentina. The abbreviation of the generic names were according to Marcondes.^14^ DNA extractions were obtained from five male specimens of *Ev. cortelezzii* and one of *Mg*. *migonei*. The genomic DNA was extracted using the rapid method described by Pinto et al.^10^ The DNA obtained was used as the template to amplify the ~658 bp fragment of the *COI* barcode region and the amplification was performed as described in Folmer et al.^15^ The polymerase chain reaction (PCR) products were electrophoresed in 1% TAE agarose gel stained with GelRed (Biotium Inc, Hayward, USA). All sequencing reactions were carried out in both directions using an ABI3730XL automatic sequencer (Macrogen Inc., Korea) with the same set of PCR primers. The sequences were edited using BioEdit v. 7.2^16^ The primer regions were removed from the sequences. Comparisons with available sequences were performed using Basic Local Alignment Search Tool (BLAST) to check for sequence homology and species identification of *Mg. migonei*. The *COI* gene sequences were aligned by nucleotides using the Muscle algorithm.^17^ The barcode sequences obtained in this study are deposited in GenBank under accession numbers: MN432906 (Cba1828M); MN432907 (Cba1825M); MN432908 (Cba1829F); MN432909 (Cba1837M); MN432910 (Cba1839M); MN432911 (Cba1856M). Pairwise nucleotide sequence divergences and the mean intra- and interspecific distances were estimated using Kimura two-parameter (K2P) distance^18^ implemented in Mega v7.^19^ Subsequently, the divergences obtained were used to generate a neighbour-joining (NJ) tree, using Mega v7 to evaluate the clustering pattern among species. The statistical support for the clusters was estimated using the Bootstrap support value (BSV) obtained from 1,000 replicates. Two mosquito sequences of *Culex bidens* (Diptera: Culicidae) were used as outgroup (GenBank numbers: KY581209 and KY581211). The automatic barcode gap discovery (ABGD) approach^13^ was employed to find barcode gaps for the primary species or OTUs delimitation, and to corroborate results obtained in the NJ topology. In this study, the default range of intraspecific divergence was limited between 0.001 and 0.1. Sequences from GenBank were obtained as references in the species delimitation analysis: *Ev. evandroi* (2), *Ev. termitophila* (4), *Expapillata firmatoi* (2), *Lutzomyia longipalpis* (6), *Mg. migonei* (5), *Pintomyia bianchigalatiae* (2), *Pi. fischeri* (4), *Pi. misionensis* (2), *Pi. monticola* (4), and *Sciopemyia sordellii* (2) from Brazil; Colombia, and Perú. These species were selected because they were recorded from Argentina and belong to the subtribe *Lutzomyiina*, as *Ev. cortelezzii* and *Mg. migonei*. We attempted to assess the degree of divergence of the first barcode sequences for *Ev. cortelezzii* and *Mg. migonei* for the science and Argentina, respectively.

Sixty-two specimens were captured and were morphologically identified as follows (Table): *Ev. cortelezzii* s.s. (n = 12), Cortelezzii complex (n = 49), and *Mg*. *migonei* (n = 1). Five males of *Ev. cortelezzii* and one of *Mg*. *migonei* were sequenced. The mean K2P sequence distance within the 13 nominal species was 1.03%. The frequency histogram of mean *COI* K2P sequence intra- and interspecific divergences may be seen in Fig. 1, showing that a clear barcoding gap was established. The NJ tree was built using the K2P distances between the specimens, and the primary species delimitation from the strict partition obtained from the ABGD analysis, as shown in Fig. 2. The ABGD analysis provided ten possible partitions for these specimens (Fig. 3). The most conservative partitioning (strict partitioning) identified 14 OTUs (Fig. 2) with values of prior intraspecific divergence between 0.17% and 3.5% because values below 0.17% or above 3.5% can over- or underestimate the number of species, respectively. The 14 OTUs mentioned were identified from 13 morphological species because one of the original groups, *Pi. fischeri*, was split into two barcode groups (Fig. 2), showing a deep intraspecific variation. The clusters enclosing *Pi. fischeri*, provisional species (PS)1 and PS2, showed group sequences from Brazil and Perú, separately. A plausible explanation is related to the historical events of vicariance, which can reduce gene flow between subpopulations of the same species, leading to speciation.^20^ Over time, morphological differentiation tends to take longer because changes in the morphological traits require changes in the multiple genes.^21^ The sequences of *Lutzomyia longipalpis* from Brazil and Colombia were considered *a priori* as the two provisional species because the species in Brazil showed a deep intraspecific variation between the regions of the country, as evidenced by Pinto et al.^10^ The previously mentioned concept has been supported by the outcomes. The sequences of *Pi. monticola* were also treated as the two provisional species (PS1 and PS2) because they belong to a cryptic species complex.^10^ The sequences of the nominal species *Mg. migonei* formed a well-supported cluster (100% BSV), with a mean intraspecific K2P distance of 2.24% and was recognised as an OTU by the ABGD partitioning (Fig. 2). It is important to note that *Mg. migonei* sequences belonging to Argentina differ from those found in Brazil and Colombia (Fig. 2). Novel sequences of *Ev. cortelezzii* were grouped in a well-supported cluster (100% BSV) and diagnosed as the primary species by the ABGD algorithm (Fig. 2). Even though all the *Ev. cortelezzii* specimens that were sequenced had been collected in Córdoba Province in Argentina, it is worth noting that they were not clustered with other congeneric species that were also considered, as shown by Pinto et al.^10^ In the latter, sequences of *Ev. carmelioni* were grouped with *Ev. lenti*, from specimens collected in the distant states of Espírito Santo and Mato Grosso in Brazil and were not recognised as species by the ABGD partitioning. In our analyses, three species of the genus *Evandromyia* from Argentina and Brazil were diagnosed as different OTUs.

**TABLE:** Information of specimens of Phlebotominae collected between March and April 2018 in Córdoba City

Species	Month	Site	Sex	N
Cortelezzii complex	March	BG	F	7
*Evandromyia cortelezzii* s.s.			M	6
Cortelezzii complex		SMUR	F	12
*Ev. cortelezzii* s.s.			M	1
*Migonemyia migonei*			M	1
Cortelezzii complex		ZG	F	1
Cortelezzii complex	April	BG	F	14
*Ev. cortelezzii* s.s.			M	4
Cortelezzii complex		SMUR	F	14
*Ev. cortelezzii* s.s.			M	1
Cortelezzii complex		ZG	F	1

BG: bajo grande sewage treatment plant of Córdoba municipality; F: female; M: male; SMUR: San Martín Urban Reserve; ZG: zoo garden.

**Fig. 1:**
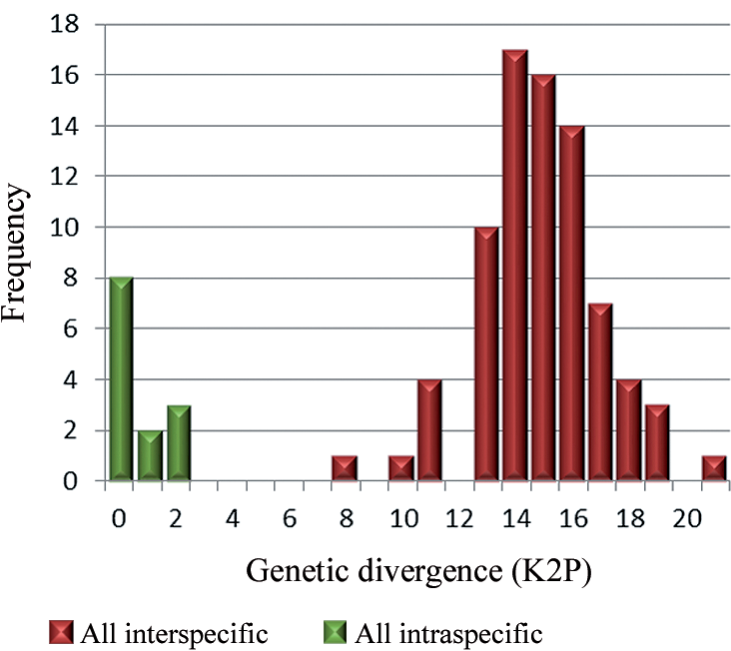
frequency distribution of intraspecific and interspecific pairwise genetic divergence of the specimens belonging to the subtribe *Lutzomyiina* from Argentina, Brazil, Colombia, and Perú. Pairwise genetic distances were calculated using Kimura’s two parameter (K2P) distance.

**Fig. 2:**
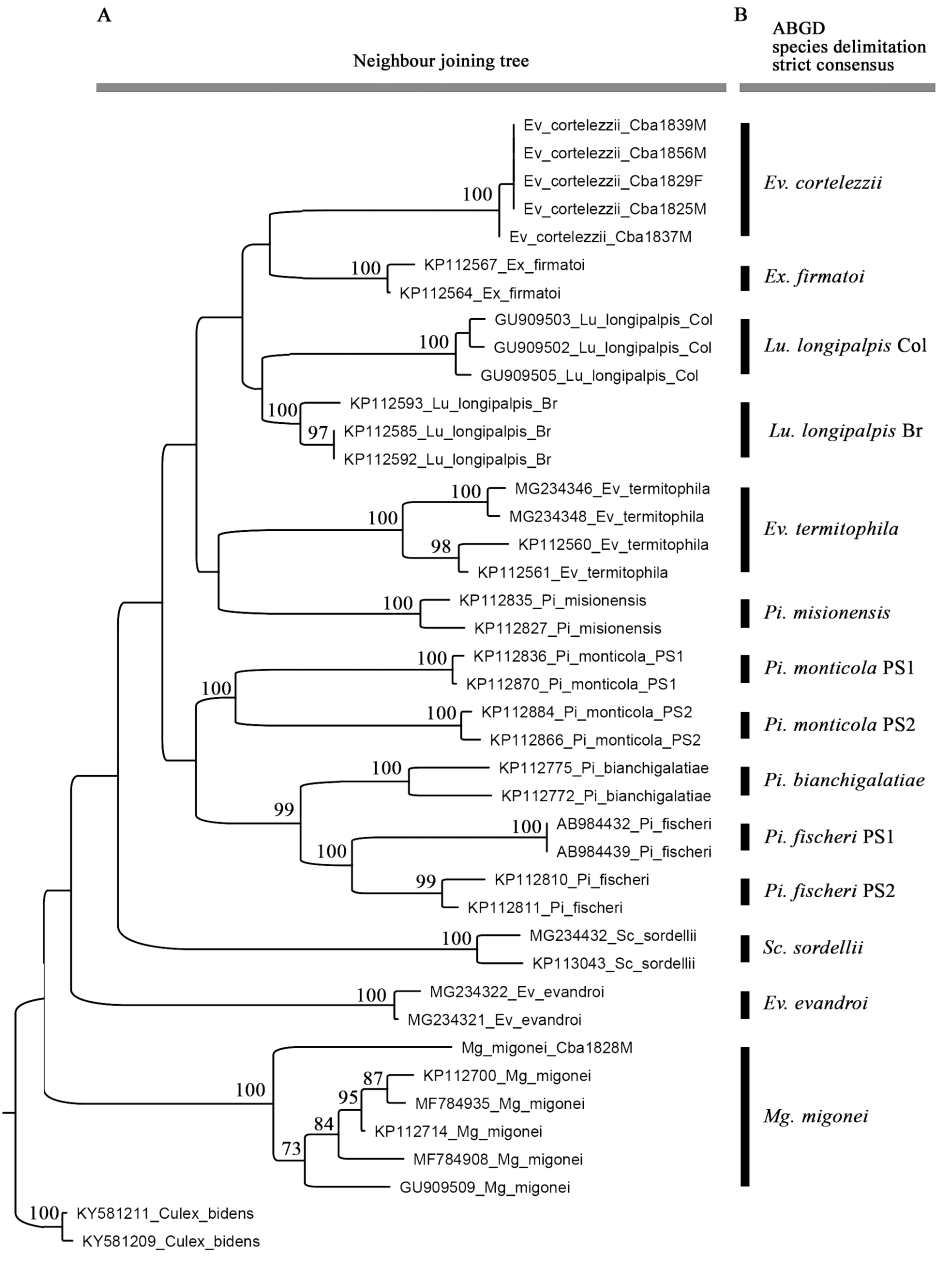
neighbour-joining tree and species delimitation analysis of 41 cytochrome *c* oxidase subunit I (*COI*) sequences generated from specimens belonging to the subtribe *Lutzomyiina* from Argentina, Brazil, Colombia, and Perú. (A) neighbour-joining tree. Numbers at branches indicate bootstrap support value (≥ 70%); (B) automatic barcode gap discovery (ABGD) analysis.

**Fig. 3:**
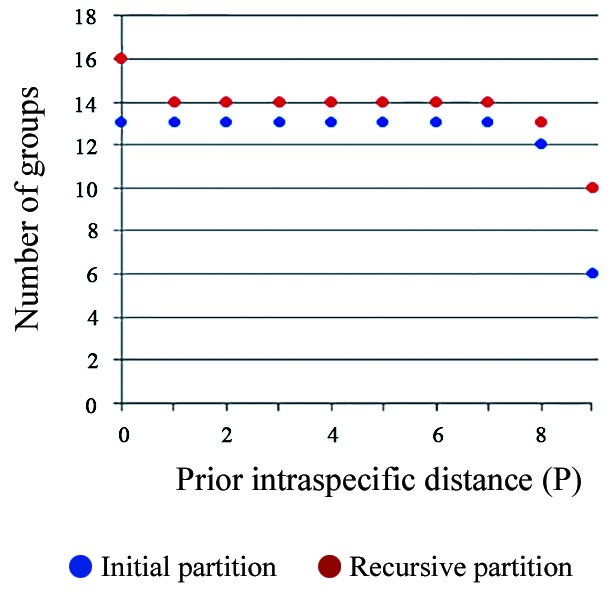
number of groups among the 39 specimens of sand flies from Argentina, Brazil, Colombia, and Perú based on the values of prior intraspecific divergence found by the automatic barcode gap discovery (ABGD) software as potential barcode gaps using a range of 0.001 to 0.1 for prior intraspecific divergence.

*Mg. migonei* and *Ev. cortelezzii* are widely distributed in Argentina,^3^ both species being important in public health. *Mg migonei* is associated with the agents of visceral Leishmaniasis (VL) and TL, *Leishmania braziliensis*, and *L. infantum*, respectively,^22^ ^,^ ^23^ and was considered the putative vector of VL in Santiago del Estero Province.^22^ Female specimens of the Cortelezzii complex were found naturally infected with *L. braziliensis* in Chaco Province.^24^

The first sequences of *COI* barcode region are now available for sand flies for Argentina. The sequence of *Mg. migonei* in its southernmost known distribution and the sequences of *Ev. cortelezzii* are the first for the species and allow the molecular identification of the females of the complex. Further studies should be carried out to obtain *COI* sequences from the males of *Ev. sallesi* and to solve identification problems of females of the complex. These sequences are useful tools for the future molecular identification of these species in other locations in the New World.
